# Supplementary material for: Development of a novel Bruton’s tyrosine kinase inhibitor that exerts anti-cancer activities potentiates response of chemotherapeutic agents in multiple myeloma stem cell-like cells
Source: Front Pharmacol. 2022 Sep 9;13:894535. doi: 10.3389/fphar.2022.894535 (PMC9500300; doi:10.3389/fphar.2022.894535)
Supplement: Supplementary file 1 [file Presentation1.PPTX]

## Slide 1
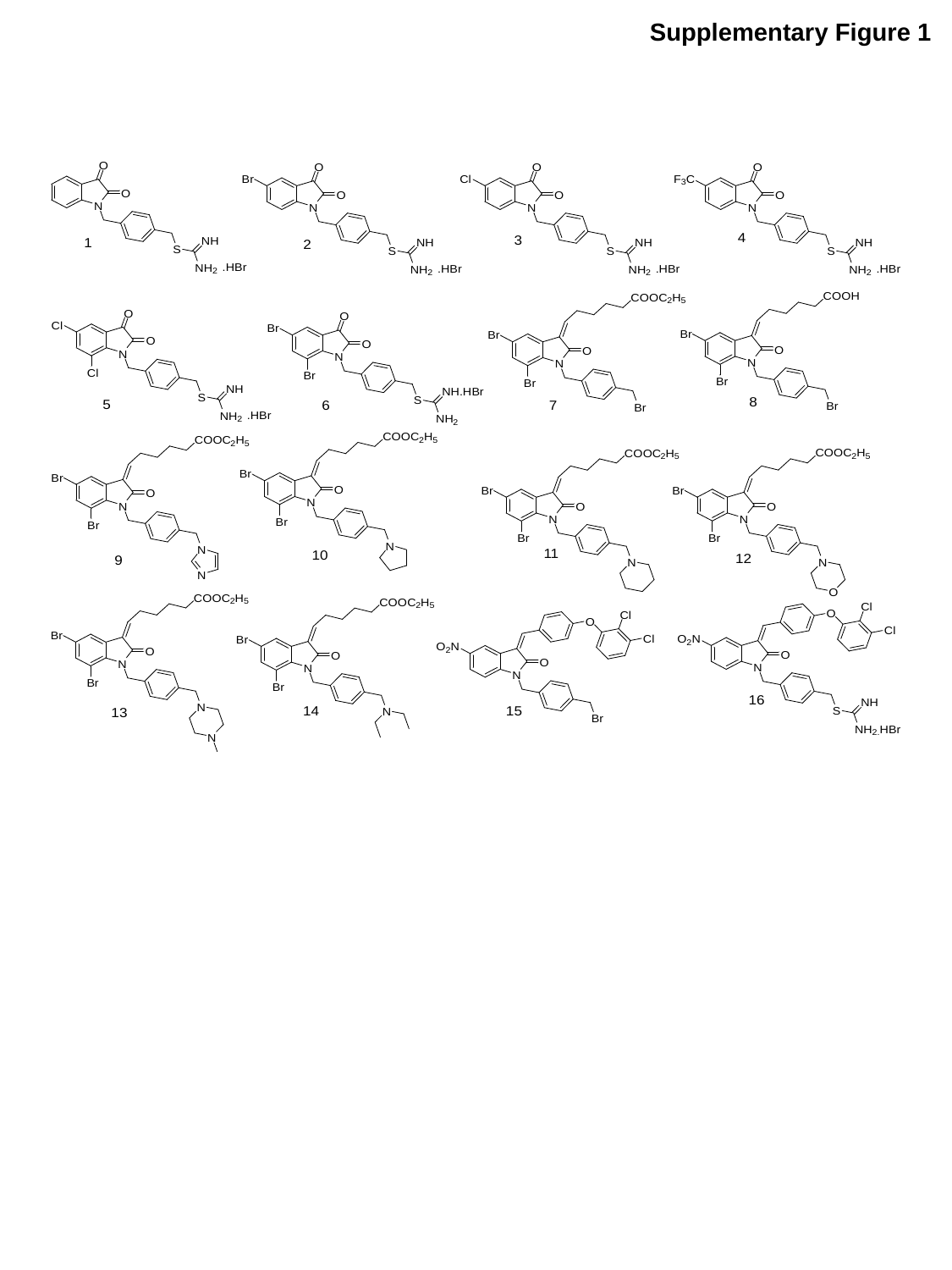

Supplementary Figure 1

## Slide 2
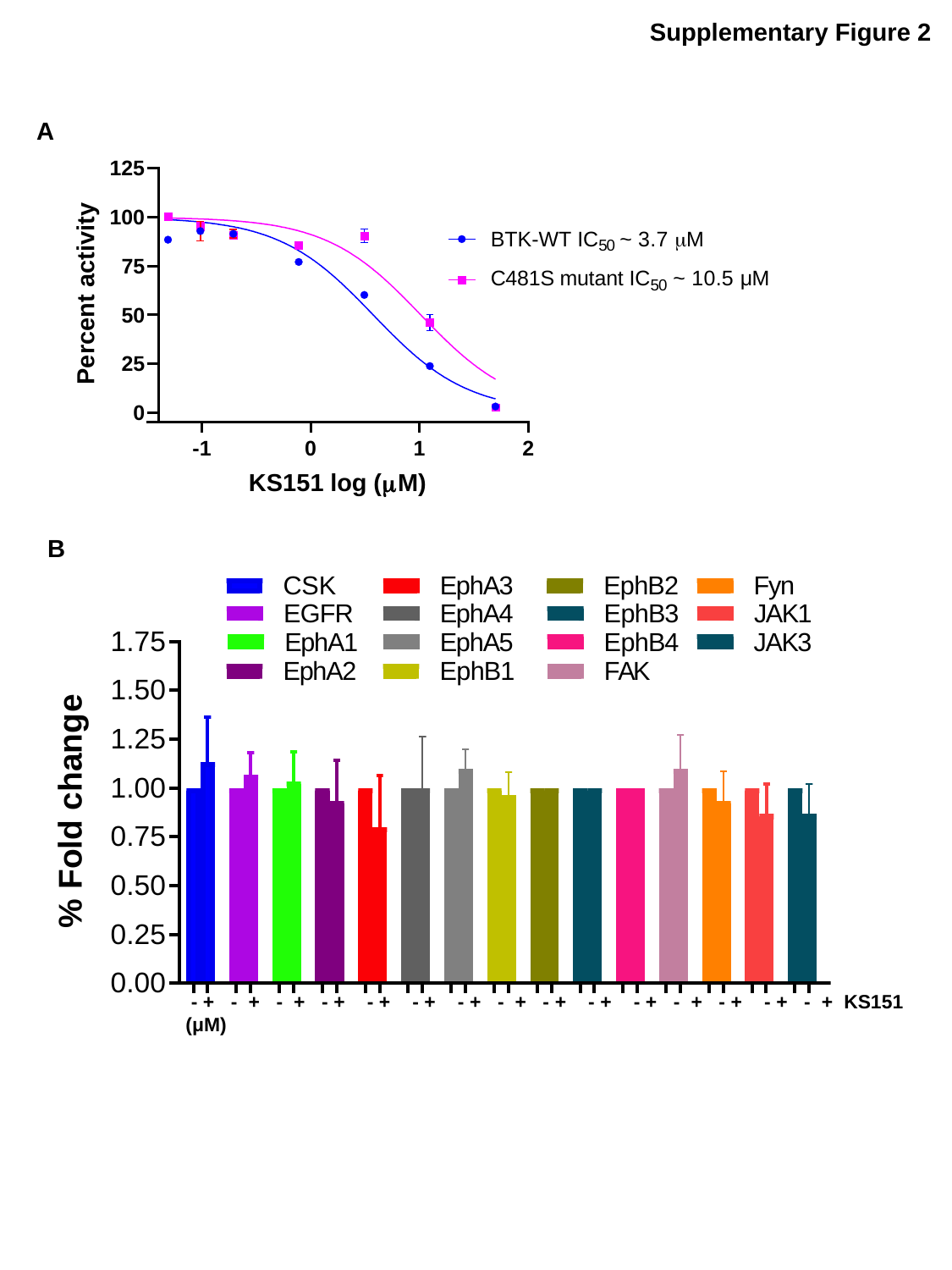

Supplementary Figure 2
A
B
 - + - + - + - + - + - + - + - + - + - + - + - + - + - + - + KS151 (μM)

## Slide 3
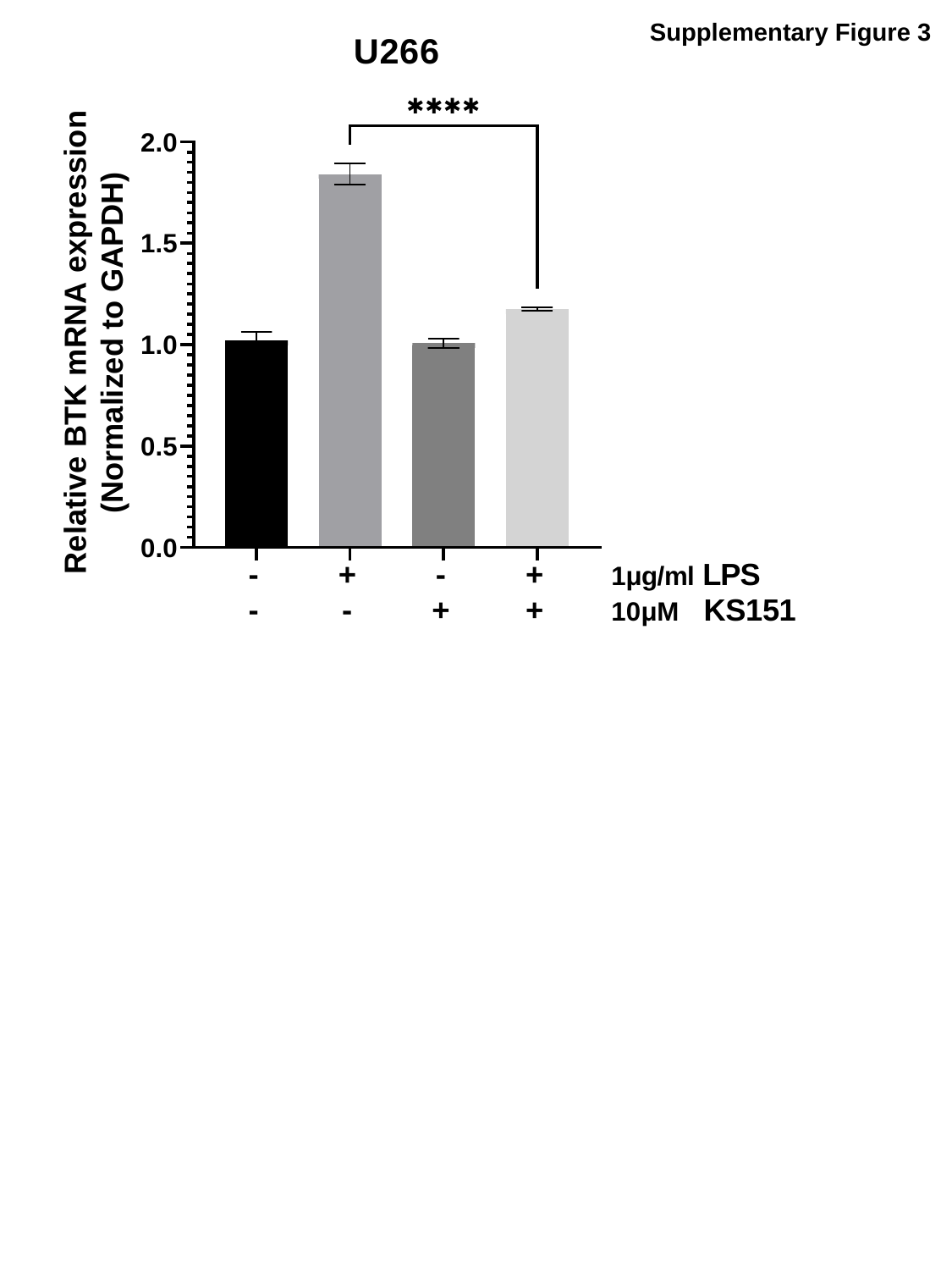

Supplementary Figure 3

## Slide 4
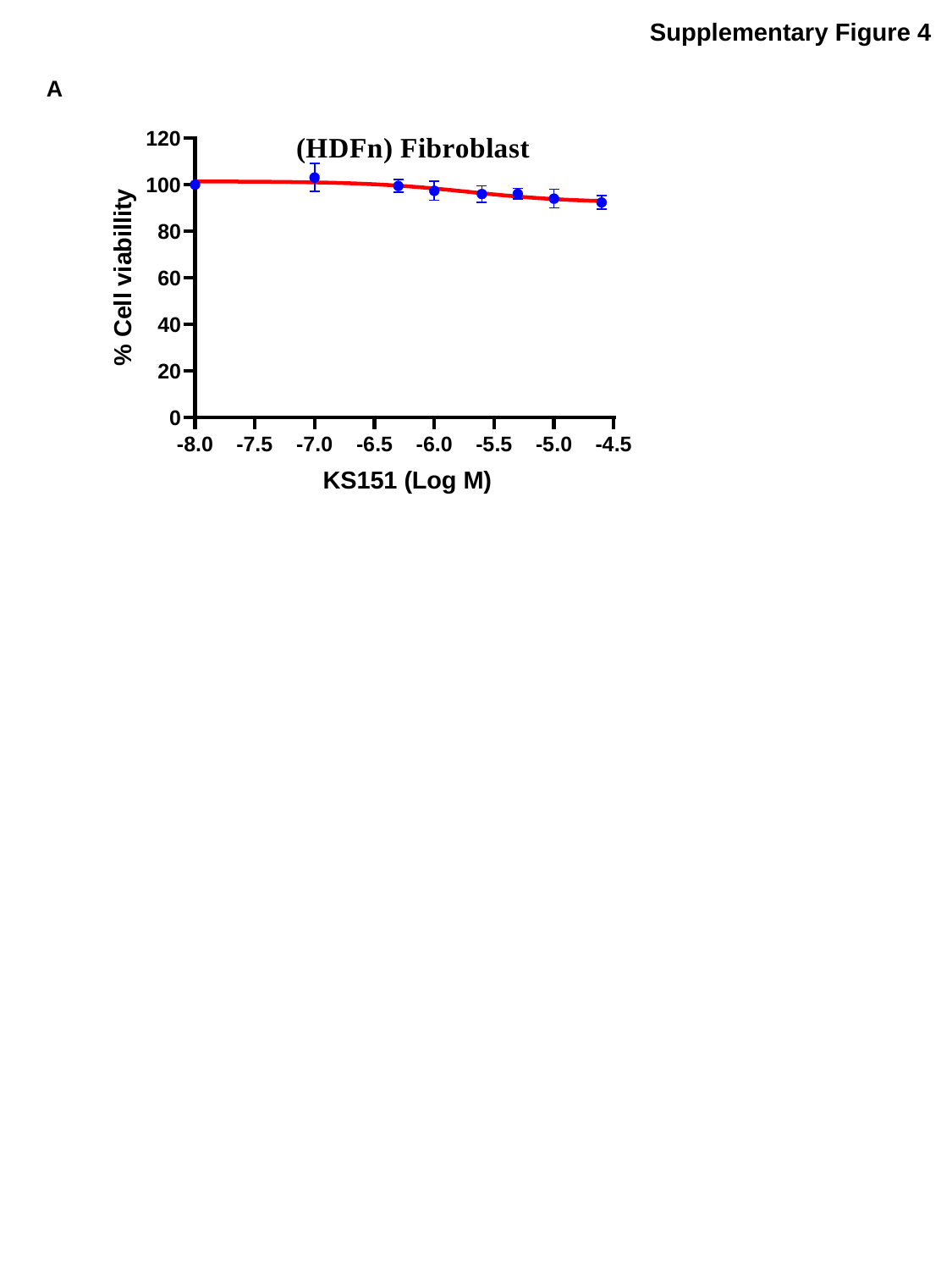

Supplementary Figure 4
A

## Slide 5
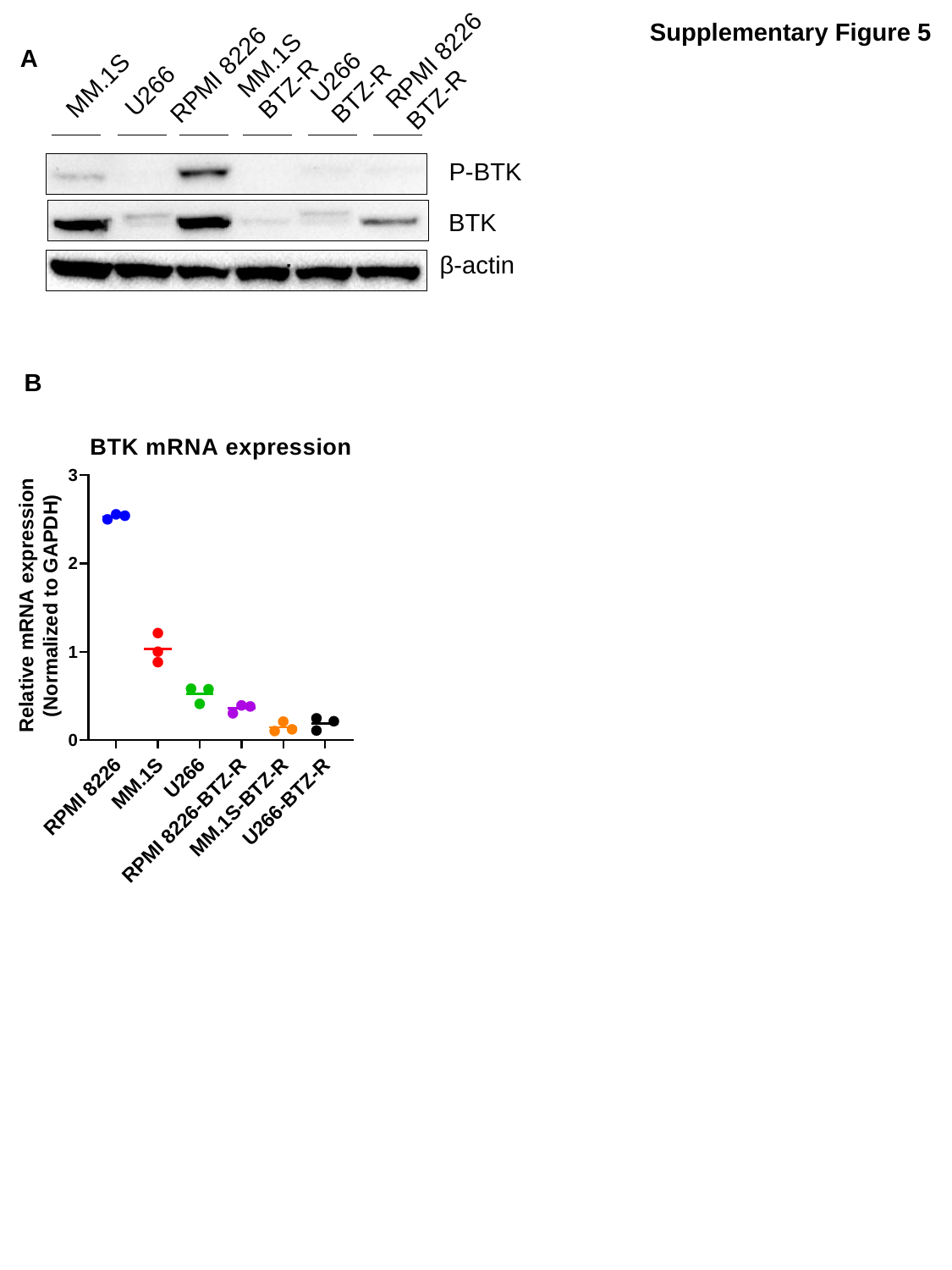

Supplementary Figure 5
MM.1S BTZ-R
RPMI 8226 BTZ-R
U266 BTZ-R
P-BTK
BTK
β-actin
A
RPMI 8226
U266
MM.1S
B

## Slide 6
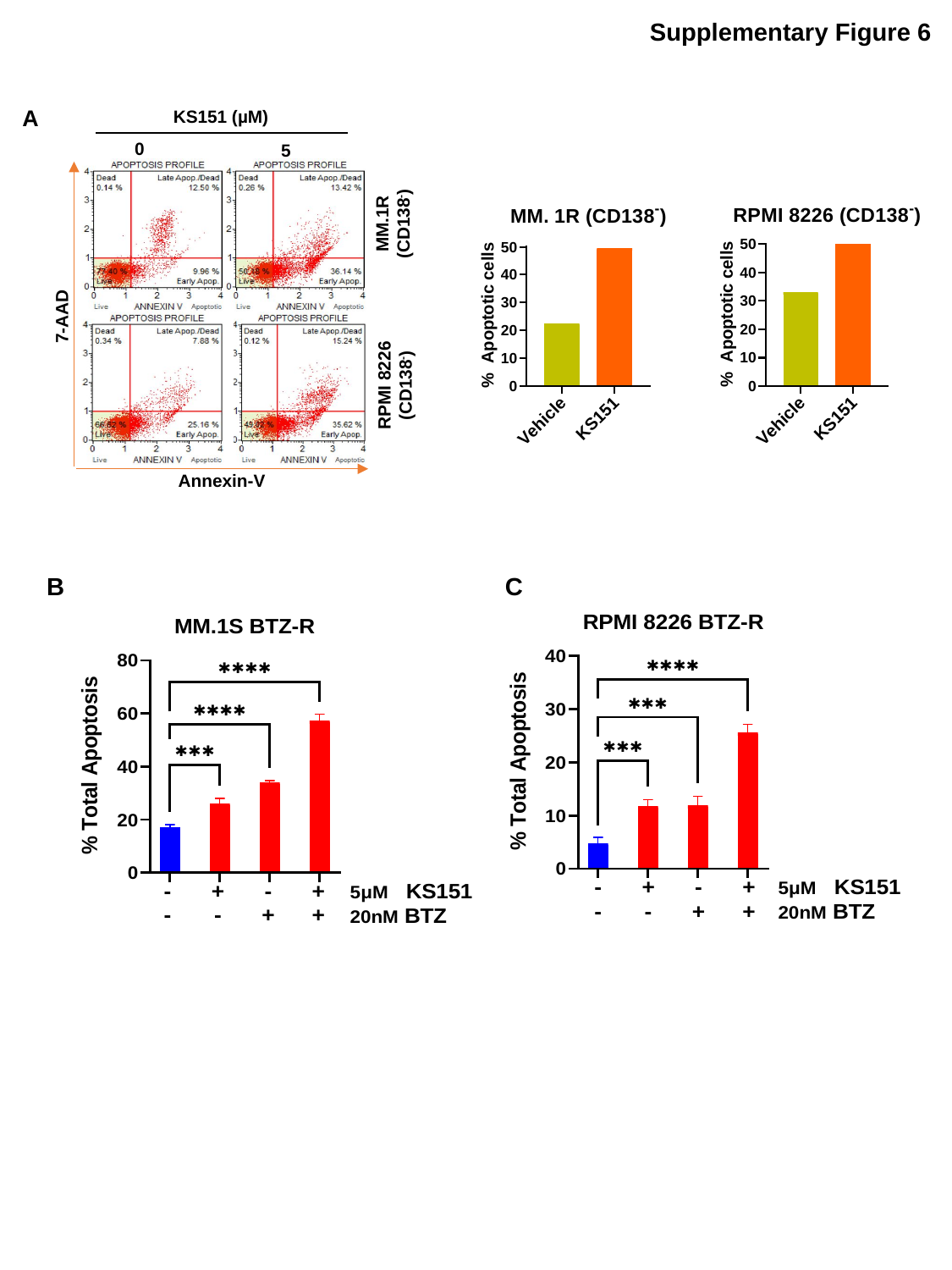

Supplementary Figure 6
A
KS151 (µM)
0
5
MM.1R
(CD138-)
7-AAD
RPMI 8226
(CD138-)
Annexin-V
B
C

## Slide 7
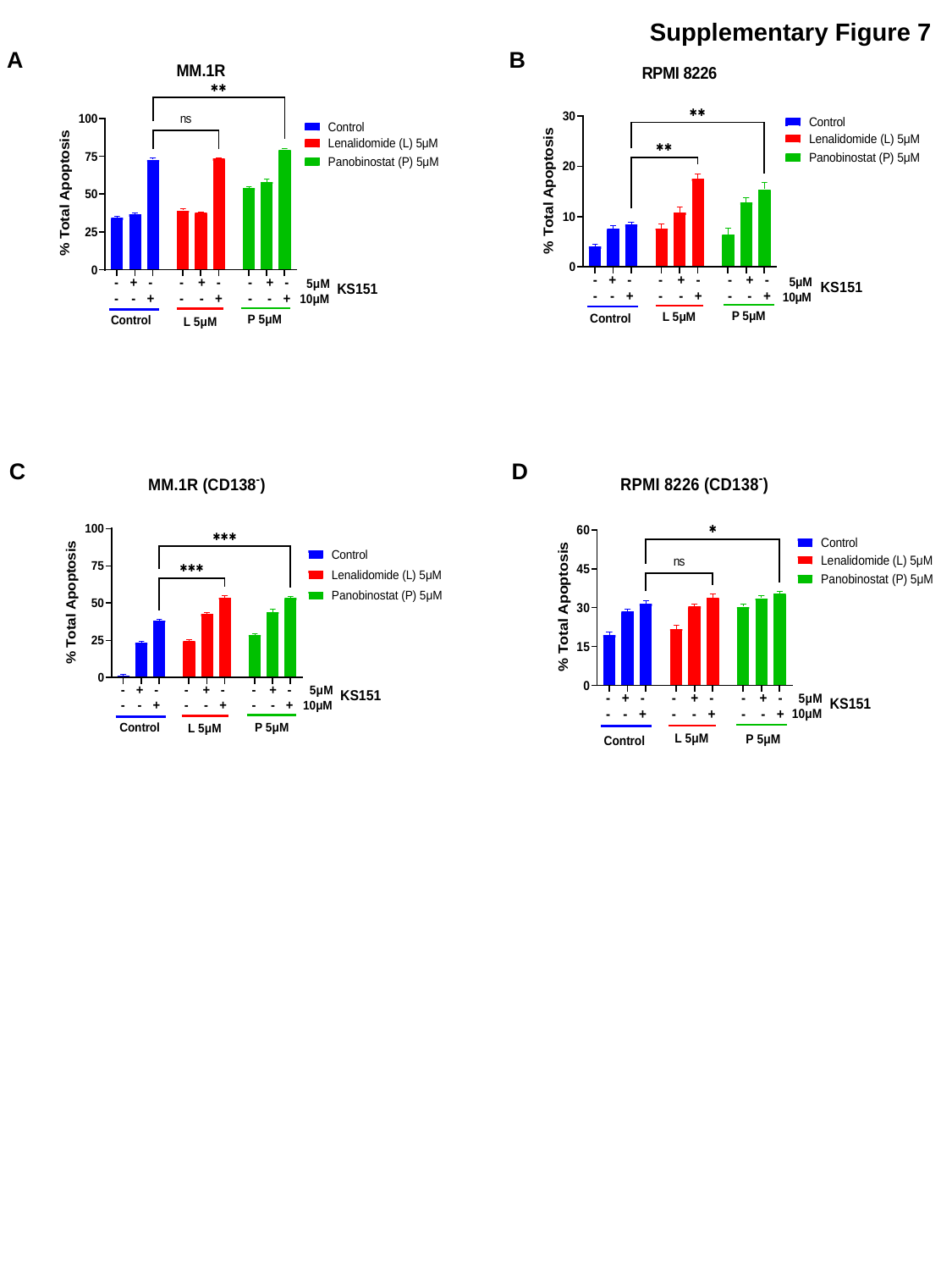

Supplementary Figure 7
A
B
C
D
